# Long-term survival of 2997 finger metacarpophalanageal joint arthroplasties from the Norwegian Arthroplasty Register

**DOI:** 10.1177/17531934221129961

**Published:** 2022-11-02

**Authors:** Eirik S. Brendsdal, Stein A. Lie, Ove Furnes, Leiv M. Hove, Yngvar Krukhaug

**Affiliations:** 1 The Norwegian Arthroplasty Register, Department of Orthopedic Surgery, Haukeland University Hospital, Bergen, Norway; 2 Department of Clinical Medicine, University of Bergen, Bergen, Norway; 3 Department of Clinical Dentistry, University of Bergen, Bergen, Norway

**Keywords:** Finger MCP joint arthroplasty, long-term survival, register study, Swanson, Avanta, Neuflex, Ascension, MCS

## Abstract

We present the long-time survival of 2997 primary metacarpophalangeal (MCP) joint implants from the Norwegian Arthroplasty Register from 1994 to 2019. Six different implants were compared in terms of survival and risk of revision. The majority of implants were inserted in patients diagnosed with inflammatory diseases and in women. The overall survival was found to be 94%, 89%, 85% and 84% after 5, 10, 15 and 20 years. The most prevalent reason for revision was a fractured prosthetic component, and the second was pain. Implants inserted in the right hand and in younger patients had a higher risk for revision. Sex, type of implant, finger treated, one- or two-component prosthesis, and inflammatory or non-inflammatory conditions did not influence the survival. The frequency of MCP joint implantations decreased during the observation period. Our data show satisfactory long-term survival of the MCP implants, with no difference found between implant types or concepts.

**Level of evidence:** II

## Introduction

Finger joint prosthesis, such as for the metacarpophalangeal (MCP) joint, have historically been used most often in patients with inflammatory joint disorders. Although the use of these prostheses is well described, there remains a lack of large studies reporting the long-term survival of MCP joint implants (Boe et al., 2018; Claxton et al., 2022; Cook et al., 1999; Notermans et al., 2020; Wagner et al., 2019). There is ongoing discussion about the ideal implant and the longevity of different types (Escott et al., 2010), due to the overall high reoperation rates, even though most surgeries do not involve revision arthroplasties (Claxton et al., 2022).

The Norwegian Arthroplasty Register (NAR) has an extensive record of patients who received MCP joint implants from 1994 to the present time. The aim of this study was to present the 20-year results and survival rates of different types of MCP joint implants and compare the outcomes of these implants.

## Methods

The NAR was established in 1987 and expanded to include all joint arthroplasties in 1994 (Furnes et al., 1996; Havelin et al., 2000). The registry aims to ensure the quality of arthroplasty surgeries at a national level and to identify inferior implants before they are used in many patients. The NAR receives registration forms directly from surgeons in all hospitals in Norway after every implant operation, with data on the following: date of operation, operated joint, operating time, perioperative complications, implant type (with product identification), operated finger (index, middle, ring and little), sex, side, age and cause of primary and revision surgery, and type of reoperation. The same form is used for revision operations (Havelin et al., 2000). Survival of the implants is defined from the date of the primary operation until the endpoint, defined as any revision operation, namely removal or exchange of the implant or addition of implant parts.

Patient death, emigration or the end of the study (31 December 2019) were also considered endpoints. Revisions were reported in relation to the primary operation by joint, side and specific finger using the unique person ID given to each Norwegian inhabitant at birth. All implants were included for the overall survival analysis. Implants used in less than six fingers were excluded when comparing survival between implant brands and models. Implants used in the index to little finger MCP joints were included in the analysis and separated in the right and left hand. We divided the patients into three age categories at the primary operation: <60 years, 60–69 years and ≥70 years. We also used age at the primary operation as a continuous variable in the regression models, with the average age for sex and the different types of MCP joint implants reported. The analysis included six different MCP implants, which we divided into two groups, ‘one-component silicone implants’ (OC), and ‘two-component metal implants’.

The diagnoses were categorized in two groups: an inflammatory group (IG) (including rheumatoid arthritis (RA), psoriatic arthritis, connective tissue disease, systemic lupus erythematosus sequela, hemochromatosis, scleroderma, Reiter’s disease/reactive arthritis, arthritis urica, Sjögren’s syndrome, crystal arthritis, granulomatosis with polyangiitis) and a non-inflammatory group (NIG) (including primary osteoarthritis (OA), sequelae after luxation, osteonecrosis, infection sequela, fracture sequelae, acute fracture, sequela after ligament damage, osteochondritis, iatrogenic joint damage, amputation sequela and haemophilia sequels). The analysis included reasons for revisions.

### Types of implants

**Figure 1.**
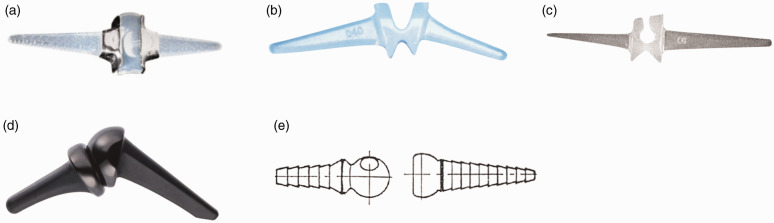
Implant types: (a) Silastic HP 100 and Silastic HP 100 II, (b) Avanta, (c) NeuFlex, (d) Ascension® MCP PyroCarbon and (e) MCS.

Six different implants were included, four one-component silicone prostheses (Silastic HP 100 Swanson Finger Joint (Wright Medical Group Company, Arlington, VA, USA) (Figure 1(a)), Silastic HP 100 II Swanson Finger Joint (Wright Medical Group Company, Arlington, VA, USA) (Figure 1(a)), NeuFlex (Johnson & Johnson Medical Ltd., Livingston, UK) (Figure 1(b)) and Avanta (Avanta Orthopaedics, San Diego, CA, USA) (Figure 1(c))), and two two-component implants (Ascension® MCP PyroCarbon Total Joint (Ascension Orthopedics, Inc. Austin, Tx, USA) (Figure 1(d)) and MCS (Modular Implant AG, Zug, Switzerland) (Figure 1(e))). See Appendix S1: Implant description, available online.

### Statistical analysis

For categorical variables, frequencies and cross-tables were used. Continuous variables were presented using mean and standard deviations. For comparisons, chi-squared tests and two-sided *t*-tests were applied. For time to revision, Kaplan–Meier probabilities were calculated. Cox regression, with robust variance estimators to account for multiple finger implants per patient, was used. Age, sex, side, if the implant had one or two components, and which MCP joint (index to little finger) were considered confounding variables and included in the adjusted Cox regression if statistically significant in the unadjusted analysis. We consider a value of *p* < 0.05 as statistically significant.

## Results

**Figure 2.**
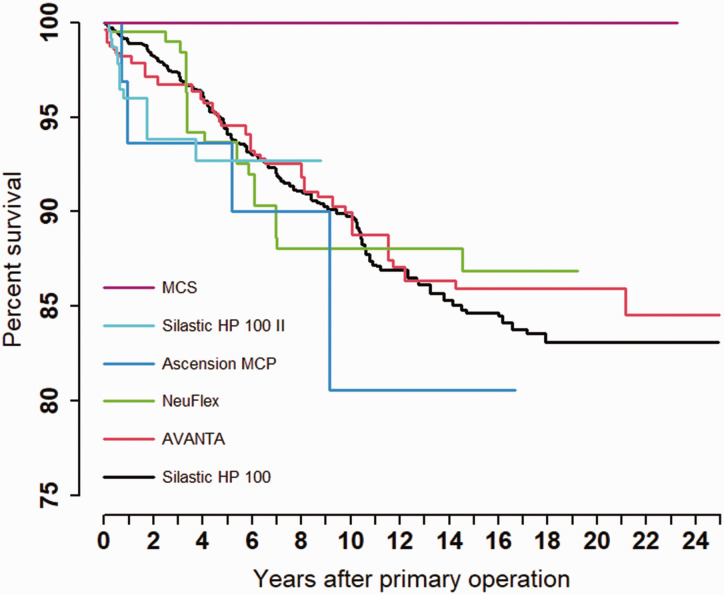
Kaplan–Meier Survival: brands and models.

A total of 3000 primary operations of MCP joint implants in 913 patients from 1994–2019 were reported. Twenty-eight Norwegian hospitals reported between one and 546 primary insertions of MCP joint prostheses. The majority were performed in women (87%). Three cases were excluded because of the use of the type of prosthesis in less than six fingers (one Moje and two SR Avanta implants). In total, 2997 implants were included in the study (Table 1). The mean age for men and women was similar, 61.9 and 61.4 years, respectively. Arthroplasty of the MCP joint was performed more commonly (60%) in the right hand. A percentage of the two most used implants, Silastic HP 100 (13%) and Avanta (12%) had a follow-up time of more than 20 years (Table 1, Figure 2). Replacements in the index finger MCP joint were most frequently reported (*n* = 899) (Table 1). The majority of the replacements were performed in patients diagnosed with inflammatory disease. Most patients had several joints replaced, and one-component implants were most commonly used (Table 1).

**Table 1.** Demographics, prosthesis survival and Cox regression.

	*N*	Revision	Survival (95% CI)				FU^b^	Cox	*p-*value
			5-year	10-year	15-year	20-year		HR (95% CI)^a^	
Prosthesis brand
Silastic HP 100	1967	229	94 (93–95)	90 (88–91)	85 (83–87)	83 (81–85)	24.9	1	Ref.
AVANTA	558	61	95 (92–96)	90 (87–92)	86 (82–89)	86 (82–89)	24.9	0.91 (0.56–1.49)	0.52
Silastic HP 100 II	234	14	92 (88–96)	–	–	–	8.7	1.67 (0.70–4.0)	0.067
NeuFlex	198	23	94 (89–96)	88 (83–92)	87 (81–91)	–	19.2	0.90 (0.42–1.92)	0.64
Ascension MCP PyroCarbon	34	5	94 (77–98)	81 (59–92)	81 (59–92)	–	16.6	1.36 (0.46–4.0)	0.50
MCS	6	0	–	–	–	–	23.2	–	–
MCP joint									
MCP index	899	105	92 (90–94)	89 (87–91)	85 (82–88)	84 (81–88)	24.8	1	Ref.
MCP middle	775	86	94 (92–96)	88 (86–91)	85 (82–88)	84 (80–87)	24.9	0.96 (0.82–1.14)	0.67
MCP ring	654	72	95 (93–97)	89 (86–92)	84 (81–88)	84 (80–87)	24.9	0.94 (0.75–1.18)	0.62
MCP little	669	69	95 (93–97)	91 (88–93)	85 (82–89)	84 (71–88)	24.9	0.88 (0.70–1.11)	0.29
MCP index-little	2997	332	94 (93–95)	90 (88–91)	85 (83–87)	84 (82–86)	24.9	–	–
Prostheses									
One-component (OC-silicone)	2957	327	94 (93–95)	90 (88–91)	85 (83–87)	84 (82–86)	24.9	1	Ref.
Two-component (TC)	40	5	94 (88–100)	84 (72–97)	84 (72–97)	84 (72–97)	23.2	1.07 (0.37–3.14)	0.90
Sex									
Male	387	31	95 (92–97)	92 (89–95)	87 (82–91)	87 (82–91)	23.8	1	Ref.
Female	2610	301	94 (93–95)	90 (88–90)	85 (83–86)	84 (82–85)	24.9	1.21 (0.66–2.23)	0.53
Age									
<60	1219	189	95 (93–96)	89 (87–91)	82 (79–84)	80 (77–83)	24.9	1	Ref.
≥60–<70	974	83	94 (92–95)	91 (88–92)	89 (87–91)	90 (87–91)	24.1	0.62 (0.41–0.94)	0.02
≥70	804	60	93 (91–95)	90 (87–92)	90 (87–92)	90 (87–92)	23.8	0.74 (0.44–0.94)	0.26
Diagnoses									
Non-inflammatory group (NIG)	105	9	94 (87–97)	93 (85–96)	91 (83–95)	87 (72–94)	23.2	1	Ref.
Inflammatory group (IG)	2874	322	94 (93–95)	89 (88–90)	85 (83–86)	84 (82–86)	24.9	1.30 (0.65–2.59)	0.46
Side									
Left	1205	102	96 (95–97)	92 (91–94)	88 (85–90)	87 (84–89)	24.7	1	Ref.
Right	1797	230	93 (91–94)	88 (86–89)	83 (81–85)	82 (79–84)	24.9	1.53 (1.08–2.17)	0.016
All implants	2997	332	94 (93–95)	89 (88–91)	85 (83–87)	84 (82–86)	24.9	–	–

CI: confidence interval; HR: hazard ratio; Ref.: reference.

^a^Unadjusted Hazard ratio (HR) estimated using Cox regression with robust variance.

^b^Follow-up in years.

**Figure 3.**
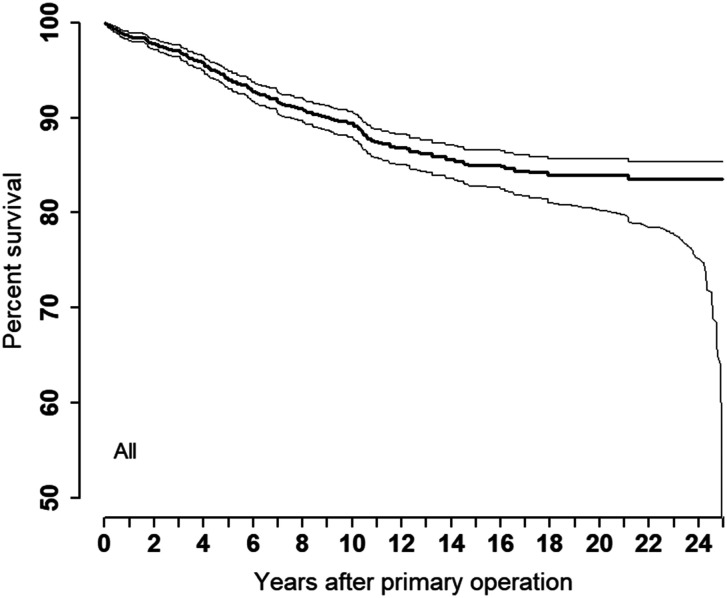
Kaplan–Meier Survival: all implants.

**Figure 4.**
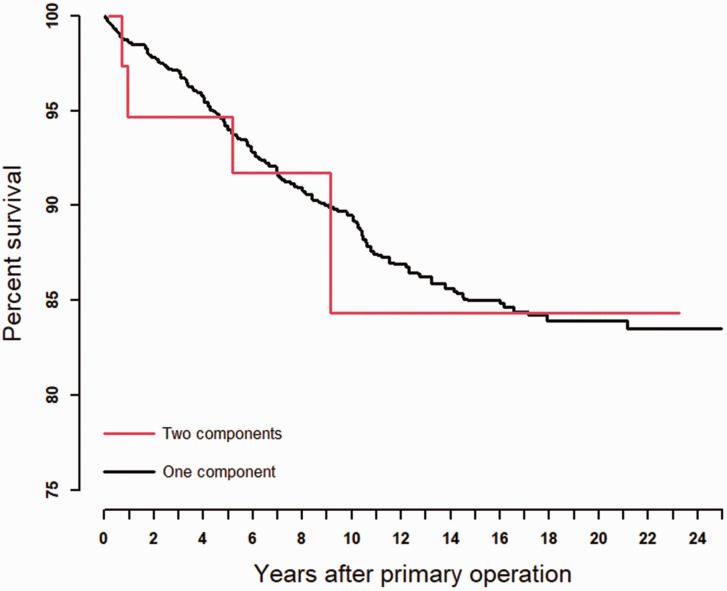
Kaplan–Meier Survival: one- and two-component implants.

The most common reasons for revision were (in descending order): fracture of the implant, pain only and instability (Table 2). The frequency of implant operations decreased during the observation period (1994–2019) (Norwegian Arthroplasy Register, 2020). The survival of all implants was 94%, 89%, 85% and 84% after 5, 10, 15 and 20 years, respectively (Table 1, Figure 3). No statistically significant differences were found when comparing different prosthesis brands (*p*-value = 0.74). No statistically significant differences in survival were found when comparing the two categories, OC-silicone and TC-metal (HR: 1.07, *p*-value = 0.896, Figure 4). The revision rate was significantly higher in the right hand compared with the left hand (HR: 1.53, *p*-value = 0.016, Table 1). Patients 60–69 years of age had a lower risk of revision (HR: 0.62, *p*-value = 0.023) compared with patients under 60 years old (Table 1). An adjusted analysis including only the significant variables (age and side) did not alter the results from the unadjusted analyses. We found no statistically significant differences in survival rate between the different MCP joints replaced. (*p*-value = 0.74, Table 1). We also found no difference in survival between NIG and IG (HR: 1.30, *p*-value = 0.457, Table 1). The survival between sexes showed no statistically significant differences (HR: 1.21, *p*-value = 0.530, Table 1).

**Table 2.** Reasons for revision.

Reason for revision	One-component	Two components
Fractured prosthetic component	134	0
Pain only	89	1
Instability	52	0
Axis error	49	0
Other	44	0
Luxation	32	0
Worn or defective	20	0
Loose distal prosthesis part	9	5
Deep infection	10	0
Bone fracture (close to the prosthesis)	10	0
Missing	2	0
Loose proximal prosthesis part	4	1
Progression of osteoarthritis	2	0
Total number of revisions	327	5

## Discussion

In this study, we looked at the survival rates of different MCP implants and found that the overall survival of the MCP implants was 94%, 89%, 85% and 84% after 5, 10, 15 and 20 years, respectively. Most implants were replaced due to a fractured prosthetic component. We did not find any differences in survival between the different implant types. Implants were most commonly inserted in the right hand, and the revision rate was higher on the right side. Younger patients had a higher incidence of revision. Most MCP joint replacements were performed in patients diagnosed with inflammatory disease. Most implants were inserted in women, and most patients had several joints replaced with one-component prostheses.

Our study included 2997 replacements in 913 patients. Boe et al. (2018) reported 5-, 10-, and 15-year survival to be 98%, 95% and 95%, respectively. Although their results were better than ours, one possible explanation is the lower number of cases (325) reported in their study. Another possible explanation is that silicone implants were used exclusively in their study, and all patients were from a single institution.

Our study included patients from all Norwegian hospitals and probably presented more of an overview. We did not show any statistically significant differences in prosthesis survival between implant types, similar to Wagner et al. (2019), who also found no difference in 5-year survival rate between pyrocarbon and silicone implants.

The most common cause of revision in our study was a fractured prosthetic component, which indicates that MCP joint silicone implants have a limited lifetime, although in terms of revision surgery, this would still be considered a long survival rate. Other studies have found that these silicone implants break after some time, but this does not always cause pain (Vahvanen and Viljakka, 1986; Wilson et al., 1993) as the implant still serves as a spacer.

Notermans et al. (2020) found no difference in survival between right and left hands, which is in contrast to our results; however, their study included a smaller number of 252 prostheses in 72 patients. Boe et al. (2018) included 325 arthroplasties in their study with a median age of 64 years (IQR 54–70). They reported no difference in rate of implant failure when comparing dominant and non-dominant hand.

In our study, we found that there was a better survival rate in older patients. A reasonable explanation may be the higher activity in younger patients. Which strengthens our result that older patients do have a better survival rate. Boe et al. (2018) investigated hazard ratio for implant failure after MCP joint arthroplasty and did not find age a risk factor. Also Cook et al. (1999) found equivalent 16-year survival rates between patients younger than 55 and patients 55 and older.

In our study, patients from the IG group constituted 97% of the replacements. Our results showed no statistically significant difference in survival between the IG and NIG. Other studies (Boe et al., 2018; Cook et al., 1999; Wagner et al., 2019) also found the great majority of implants in patients with RA or inflammatory arthritis. Claxton et al. (2022) and Notermans et al. (2020) only reported results on patients with RA and inflammatory arthritis.

Sex and the number of MCP joints replaced, showed no difference in prosthesis survival in our analysis, which is in accordance with the findings of Boe et al. (2018).

We had insufficient data to compare and highlight possible differences in reasons for revision between one and two-component prosthesis due to a low number of two-component prostheses used. Our study included 34 Ascension MCP PyroCarbon implants, and the 15-year survival was 81%. The Ontario Health Technology Assessment Series (2004) found the 16-year survival of 151 pyrocarbon MCP joint implants to be 70%. Maybe the indication for this type of prosthesis was stricter in Norway, but we cannot show this with the available data.

We found that most patients had more than one finger in each hand replaced. Other studies (Boe et al., 2018; Claxton et al., 2022; Cook et al., 1999; Kimani et al., 2009; Notermans et al., 2020; Wagner et al., 2019) have shown similar results, most probably because patients diagnosed with RA often have both hands affected.

Similar to other studies (Boe et al., 2018; Claxton et al., 2022; Wagner et al., 2019), the age of men and women was similar (61 years) at the time of joint replacement in our study.

The majority of replacements were performed in women. This is also similar to the findings in several other studies (Boe et al., 2018; Claxton et al., 2022; Kimani et al., 2009; Notermans et al., 2020; Wagner et al., 2019). One reason for this is likely that women disproportionately have RA (Favalli et al., 2019).

The frequency of total joint replacement decreased during the observation period (1994–2019). The reasons for this trend are probably the improved medical treatment options for RA leading to less need for joint replacement in these patients (Burmester and Pope, 2017; Fevang et al., 2007; Nystad et al., 2016; Sparks, 2019).

The completeness of reporting for finger prosthesis for both primary and revisionary surgery from 2008–2018 were both 57% in the NAR (Norwegian Arthroplasy Register, 2020). For the years 1999–2002, the completeness of reporting of hand procedures was 85% for primary and 76% for revision procedures compared with the Norwegian Patients Registry, which is an administrative registry (Espehaug et al., 2006). Under-reporting would affect the results only if unevenly distributed among the different prosthesis brands.

Lack of patient-reported outcomes and the missing link of the dominant and non-dominant hand may be considered a weakness in our study.

Survival estimates could be biased. This could be a particular problem for patients with RA since these may tolerate fewer implants that are not functioning well. If at time of censoring (i.e. death or emigration) the implant had failed but had not been revised, the survival estimates will underestimate the true revision rate (Murray et al., 1997).

## Supplementary Material

Supplementary material
