# Supplementary material for: Long-term survival of 2997 finger metacarpophalanageal joint arthroplasties from the Norwegian Arthroplasty Register
Source: J Hand Surg Eur Vol. 2022 Nov 2;48(1):3–9. doi: 10.1177/17531934221129961 (PMC9773006; doi:10.1177/17531934221129961)
Supplement: Supplementary material [file sj-pdf-1-jhs-10.1177_17531934221129961.pdf]

Implant description:

Silastic HP 100 Swanson Finger Joint (Wright Medical Group Company, Arlington, USA) is a flexible one-piece, intramedullary stemmed implant made of HP 100 silicone elastomer, and available in eleven sizes. The proximal stem fits into the intramedullary canal of the metacarpal phalanx, and the distal stem fits in the intramedullary canal of the proximal phalanx. Inserted with uncemented technique. In selected cases it is possible to attach a grommet, a thin titanium shield to the Silastic HP 100 Swanson Finger Joint. Figure a.

Silastic HP 100 II Swanson Finger Joint (Wright Medical Group Company, Arlington, USA) is a flexible one-piece, intramedullary stemmed implant made of silicone elastomer, and available in eleven sizes. The proximal stem fits into the intramedullary canal of the metacarpal phalanx, and the distal stem fits in the intramedullary canal of the proximal phalanx. Inserted with uncemented technique. In selected cases it is possible to attach a grommet, a thin titanium shield to the Silastic HP 100 II Swanson Finger Joint. As far as we know, only small changes were done compared to the original design of Silastic HP 100**.** Figure a.

Avanta (Avanta Orthopaedics, San Diego, CA) is a double stem one-piece implant constructed of Silflex II elastomers, made from a silicone rubber material. The proximal stem fits into the intramedullary canal of the metacarpal, and the distal stem fits in the intramedullary canal of the proximal phalanx. Inserted with uncemented technique. The Avanta MCP implant is available in seven sizes. Figure c.

NeuFlex (Johnson & Johnson Medical Limited PO BOX 1988, Simpson Parkway, Livingston, West Lothian, EH54 0AB, United Kingdom) is a one-piece double stem

implant and it is an Analsil silicone device. The proximal stem fits in to the intramedullary canal of the metacarpal, and the distal stem fits in the intramedullary canal of the proximal phalanx. Inserted with uncemented technique. The NeuFlex MCP implant is available in seven sizes. Figure b.

Ascension® MCP PyroCarbon Total Joint (*Ascension Orthopedics, Inc.*8700 Cameron Road Austin, Texas 78754) is a two-piece implant made of Pyrocarbon. Pyrocarbon is a durable and biocompatible material that has been used in orthopedics for several years. Inserted with uncemented technique. The distal part has a concave articulation surface, and the proximal part has a convex articulation surface. The implant is available in five different sizes. Figure d.

MCS (prox/dist) (Modular Implant AG, Sihlbruggstrasse 105 postfach 2261) is a two-piece implant made of forged Ti6AI4V, the surface on the anchoring shaft of porous hydroxylapatite ceramic and the joint surfaces of TiNb ceramic. Inserted with uncemented technique. The proximal part of the MCS MCP implant is available in four sizes and the distal part in tree sizes. Figure e.
